# Genome Wide Nucleosome Mapping for HSV-1 Shows Nucleosomes Are Deposited at Preferred Positions during Lytic Infection

**DOI:** 10.1371/journal.pone.0117471

**Published:** 2015-02-24

**Authors:** Jaewook Oh, Iryna F. Sanders, Eric Z. Chen, Hongzhe Li, John W. Tobias, R. Benjamin Isett, Sindura Penubarthi, Hao Sun, Don A. Baldwin, Nigel W. Fraser

**Affiliations:** 1 Department of Microbiology, Perelman School of Medicine, University of Pennsylvania, Philadelphia, PA, 19104, United States of America; 2 Department of Chemical Pathology, The Chinese University of Hong Kong, Li Ka Shing Institute of Health Sciences, Hong Kong SAR, China; 3 Penn Molecular Profiling Facility, Perelman School of Medicine, University of Pennsylvania, Philadelphia, PA, 19104, United States of America; 4 Pathonomics LLC, Philadelphia, PA, 19104, United States of America; 5 Department of Biostatistics and Epidemiology, Perelman School of Medicine, University of Pennsylvania, Philadelphia, PA, 19104, United States of America; University of Regensburg, GERMANY

## Abstract

HSV is a large double stranded DNA virus, capable of causing a variety of diseases from the common cold sore to devastating encephalitis. Although DNA within the HSV virion does not contain any histone protein, within 1 h of infecting a cell and entering its nucleus the viral genome acquires some histone protein (nucleosomes). During lytic infection, partial micrococcal nuclease (MNase) digestion does not give the classic ladder band pattern, seen on digestion of cell DNA or latent viral DNA. However, complete digestion does give a mono-nucleosome band, strongly suggesting that there are some nucleosomes present on the viral genome during the lytic infection, but that they are not evenly positioned, with a 200bp repeat pattern, like cell DNA. Where then are the nucleosomes positioned? Here we perform HSV-1 genome wide nucleosome mapping, at a time when viral replication is in full swing (6hr PI), using a microarray consisting of 50mer oligonucleotides, covering the whole viral genome (152kb). Arrays were probed with MNase-protected fragments of DNA from infected cells. Cells were not treated with crosslinking agents, thus we are only mapping tightly bound nucleosomes. The data show that nucleosome deposition is not random. The distribution of signal on the arrays suggest that nucleosomes are located at preferred positions on the genome, and that there are some positions that are not occupied (nucleosome free regions -NFR or Nucleosome depleted regions -NDR), or occupied at frequency below our limit of detection in the population of genomes. Occupancy of only a fraction of the possible sites may explain the lack of a typical MNase partial digestion band ladder pattern for HSV DNA during lytic infection. On average, DNA encoding Immediate Early (IE), Early (E) and Late (L) genes appear to have a similar density of nucleosomes.

## Introduction

Herpes simplex virus (HSV-1) is a large double stranded DNA virus (152kb genome). On infecting a host it forms lytic infections in epithelial tissue, from which it can spread to the innervating sensory neurons of the peripheral nervous system where it can establish a life long latent infection, from which it can be reactivated to cause recurrent disease (for review see: [1]). HSV causes much disease in the human population from the common cold sore to devastating encephalitis and is especially troublesome in immune-compromised individuals.

DNA viruses protect their genomes in compact protein structures called capsids. To package viral DNA into these capsids they condense it by neutralizing its charge with negatively charged proteins—histones or protamines. Small polyoma and SV40 virions have been shown to contain nucleosomes in their capsids [2,3]. However, larger Adenovirus does not have nucleosomes in its capsid, but acquires them during lytic infection [4]. Although small DNA viruses contain nucleosomes in their capsids, HSV, like Adenovirus, does not [5,6]. HSV DNA in virions has its charge partly neutralized by polyamines [5]. Following infection of tissue culture cells and entry of the cell nucleus, nucleosomes are deposited on HSV DNA within 1 hour [7], at which time the linear viral genome is circularized and gene expression initiated [8].

Nucleosomes can be deposited on newly replicated DNA by a replication coupled process [9]. However this process, which occurs about 4h into an HSV infection cycle, cannot account for nucleosomes deposited prior to HSV DNA replication. It is more likely that either the cellular DNA repair machinery used to circularize the genome, or the transcriptional machinery of the cell used to transcribe viral genes, deposits nucleosomes on the viral DNA at early times. It is also possible that a fundamental defense mechanism is depositing nucleosomes on the polyamine coated viral DNA entering the cell nucleus. Nucleosomes deposited during DNA replication contain histone variant H3.1 and nucleosomes deposited during transcription contain histone H3.3. The nucleosomes deposited early during HSV replication have been shown to contain histone H3.3, suggesting a replication independent mechanism of addition [10].

A characteristic of chromatinized DNA is the 200bp band ladder seen on gel electrophoresis following partial micrococcal nuclease (MNase) digestion. Interestingly, the nucleosomes deposited on HSV DNA during lytic infection do not provide this characteristic partial MNase digestion pattern. Instead a smear is obtained [11,12], which resolves to a mono-nucleosome band on complete digestion [7]. Thus, it seems that the distribution of nucleosomes is not regularly patterned like cellular nucleosomes. Interestingly, cell DNA has been shown to initially coat with nucleosomes in an irregular pattern which is later made uniform by ATP dependent chromatin remodeling enzymes [13]. Chromatin immuno-precipitation (ChIP) experiments suggest that only a fraction of the viral DNA is in a nucleosome protected form [14]. The partial, or lack of saturating, coverage of nucleosomes may explain the lack of a classic ordered partial MNase digestion pattern.

There are several reviews on the role of chromatin in the lytic and latent cycle of HSV infection [15,16,17,18]. Modification of histones on the viral genome is thought to promote expression of viral genes and replication of the viral genome. Cellular proteins such as cellular transcription factor (HCF-1) are important, not only for activating viral gene expression, but also in viral DNA replication, mediated through chromatin modifying enzymes [19,20,21]. Viral proteins are also thought to be involved in regulating viral gene expression through histone modification[22,23]. Location within the cell nucleus has also been shown to affect viral histone modification and thus chromatin structure [24].

In this study we have examined the distribution of nucleosomes on the HSV genome during a lytic infection of tissue culture cells at 6hr Post Infection (PI)—a midway point in the growth of the virus, when all of the genes are expressed and DNA replication has started [6]. The data show that at this time there are many fewer than the theoretical maximum number of nucleosomes present on the HSV-1 genome (60–80%). Nucleosomes on the viral genome appear to be clustered within genes and there are nucleosome free or depleted regions (NFR or NDR) not only in the gene promoter regions, but also in the coding regions and the 3’ end of genes. Furthermore, there is a trend for IE genes to have a lower density of nucleosomes compared to Early or Late genes, though we do not have the statistical power to show significance. The joint region of the genome (the junction between the ends of the repeated sequences) was found to be devoid of nucleosomes, as were the origins of DNA replication. The paucity of nucleosomes on the genome, and the clustering of those that there are, explains the lack of classical MNase partial digest ladder seen on digestion of HSV-1 chromatin from a lytic infection.

## Materials and Methods

### Cells and viruses

SY5Y cells (human neuroblastoma cell line) were grown in RPMI 1640 medium supplemented with 10% fetal calf serum and penicillin (150u/ml) and streptomycin antibiotics (150u/ml). HSV-1 strain F was used to infect these cells.

### Viral infection and virion DNA preparation

Two 175 cm^2^ flasks of SY5Y cells at 100% confluence were infected with HSV-1 at a moi of 5 PFU per cell. Virion DNA was extracted by using previously described methods (Oh and Fraser, 2008). The purified virion DNA was then fragmented by sonication for 5 min (30Sec/30Sec) with a Bioruptor (Diagenode).

### Preparation of Nucleosome Protected DNA

Cells from two 175 cm^2^ flasks were harvested by scraping, re-suspended in 10ml RSB (10mM Tris, pH 7.5, 10mM 5M NaCl, and 3mM MgCl_2_), and incubated for 5min on ice. Following addition of 10ml 1%NP40 (in RSB), the cells were mixed, incubated for 2 min on ice, overlaid with 10ml of 0.33M sucrose (in RSB), and centrifuged at 1700rpm for 10min at 4’C in a AllegraX-22R Beckman Coulter centrifuge. The pelleted nuclei were re-suspended in 1ml of MNase buffer (20mM 1M Pipes, pH7, 1mM MgCl_2_, 10mM NaCl, 1mM CaCl_2_, 5mM 2-mercaptoethanol, 0.25M sucrose, and 0.1mM PMSF). Pelleted nuclei were digested with Nuclease S7 Micrococcal nuclease (MNase)(Roche) at 5U/μl of sample for complete digestion, added 12mM EDTA to stop reaction on ice, and treated with 100ug/ml of proteinase K for overnight at 37’C. DNA was purified by a Wizard DNA clean-up kit (Promega) according to the manufacturer’s instructions. MNase digested DNA were checked by running on 1.5% low melting point (LMP) agarose gel and 150bp bands were extracted by a QIAEX II gel extraction kit (Qiagen) according to the manufacturer’s instructions.

### Array Design

The high-definition comparative genomic hybridization (HD-CGH) microarray contains a custom probe set designed using Agilent eArray tools and was manufactured by Agilent SurePrint oligomer synthesis (Agilent Technologies, Santa Clara, CA). Each HD-CGH glass slide contained eight replicate microarrays of 6091 50mer DNA probes covering the entire sequence of HSV-1 (strain 17) with overlapping, tiled probes offset by 25bp and printed in duplicate. Probes for ten Alien sequences, not present in human or HSV1- genomes, were included as controls.

### Sample Labeling and Hybridization

Five hundred ng of 150bp DNA were labeled with Cy3 dye (SureTag Complete DNA Labeling Kit, direct labeling option, Agilent #5190–4240) and assayed on arrays using CGH protocols for hybridization and washing (Oligo aCGH/CHiP Hybridization Kit and Wash Buffer Kit, Agilent #5188–5220 and #5188–5226, no Cot-1 DNA). The following protocol modifications were used for hybridization: hybridization buffer contained 30% de-ionized formamide, hybridization was carried out at 60°C, and the final wash solution was heated to 60°C. All other steps were carried out according to the manufacturer’s instructions.

Southern blotting and hybridization was performed as previously described [25].

### Image Acquisition and Data Extraction

Microarray images were acquired using an Agilent Technologies High Resolution-C Scanner. Data extraction was carried out using Agilent Feature Extraction software, version 9.5.3. Scanning and data extraction were conducted according to the manufacturer’s instructions to produce a raw signal for each probe that incorporates background pixel correction and averaging of duplicate features.

### Data normalization and peak calling

The raw signal was first processed by “backgroundCorrect” and “normalizeBetweenArrays” functions in the limma package in R/Biocoductor. The replicated arrays were then merged into one by using the median signal of probes of the replicates. Next, we removed the poorly performing probes, which were defined as the probes with log2(vir10)-log2(vir0.1)<1, where vir is signal from purified HSV-1 DNA assayed at 10ng or 0.1ng input. We then smoothed the signal by averaging the signals with a sliding window of 5 probes. To normalize for differences in probe hybridization efficiencies, the signal of each probe was adjusted to S_norm_ = [log2(test)-log2(mock)] / [log2(vir10)—log2(mock)], where test is the hybridization signal from an infected cell culture sample. To deal with outliers, we defined the maximum value of the probe signal as three median absolute deviations (MAD) away from the median and for any probe with signal beyond the maximum value, we set this signal value as the maximum value.

We next identified the histone binding regions, which show stronger binding intensities than the background. Specifically, we modeled the observed signals using a mixture of two normal components with different means and variances, one indicating histone binding, the other indicating background. The component with a larger estimated mean is considered as the histone binding and probes with signal larger than this estimated mean are defined as significant probes. We further defined the histone binding regions as those with five consecutive significant probes.

### Cluster analysis method:

The hierarchical clustering algorithm implemented in Cluster 3.0 [26] was used to visualize the global profiles across all samples. The log transformed signals of all the probes were adjusted in four steps: center probes by median, normalize probes, center samples by median, normalize samples. Then, hierarchical Spearman Rank Correlation and centroid linkage were used for clustering. The results were visualized by Java TreeView (version 1.1.6r2) [27]

### ChIP assays and qPCR

F strain-infected and mock-infected Sy5y cells were processed for ChIP assay and purified immunoprecipitated DNAs were amplified by previously described methods (Oh and Fraser, 2008). Primers were designed from the published HSV-1 sequence (GenBank accession NC_001806). F = forward; R = reverse:

**ICP0 positive 1:** TGATTGCCCGTCCAGATAAAG(F) (122729–122749)

                        CAAGCTGGTGTACCTGATAGTG(R) (122851–122872)

**ICP0 negative 1:** GGGCGGGTAAGAATGGG(F) (123306–123322)

                        GGGCAGGACTTTGTGAGG(R) (123376–123393)

**ICP0 negative 2:** CTGCGTCTGAGTCAAAGGG(F) (123707–123725)

                        GGAACCCCCTGACCCTATAT(R) (123811–123830)

## Results

### Normalization, Sensitivity and Validation of array oligos

**Fig 1:**
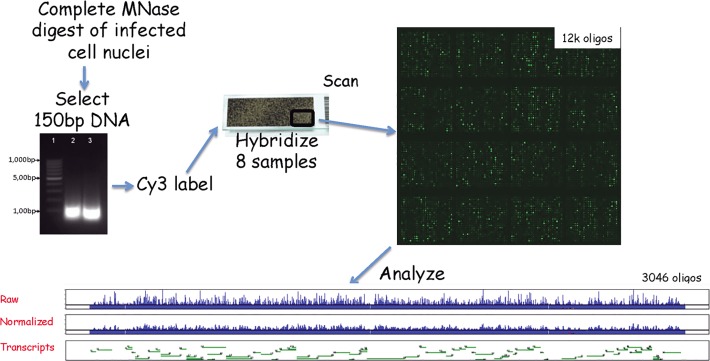
Array Design and Nucleosome Counting. SY5Y cells were infected with HSV-1 at a moi of 5. At 6h post infection cells were harvested and nuclei prepared. Nuclei were digested to completion with MNase and the DNA fragments purified. These were separated by gel electrophoresis and the 150bp band eluted and labeled with Cy3. The array slide consisted of 8 wells containing approximately 12k x 50mer oligonucleotides. The 50mers covered the entire HSV-1 genome and were tilled with a 25nt overlap. In each well there were 3 sets of 3046 oligos covering the HSV genome plus some alien (control) oligos. Eight labeled samples were hybridized on the array slide and subsequently analyzed for the position of nucleosomes on the HSV genome as described in the Methods.

**Fig 2:**
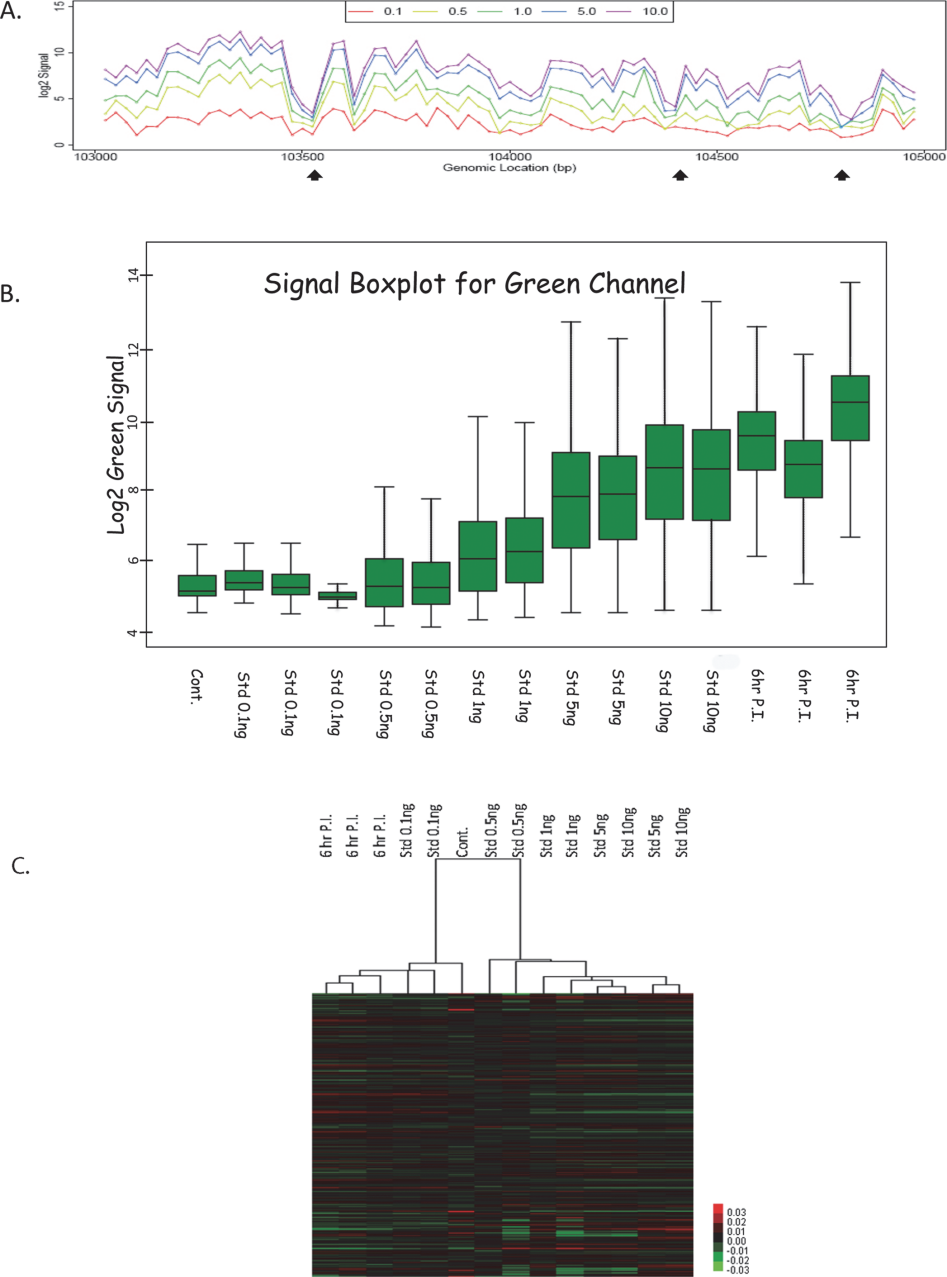
Array Validation with virion DNA, Box plot, and Heat Map Hierarchical Cluster Analysis. **A**. Section of array data with virion DNA probe is shown to illustrate the increase in signal with increasing viral DNA. Arrays were hybridized with samples of HSV-1 DNA purified from virion particles. Each sample consisted of 500ng cell DNA to which was added 0ng (control), 0.1ng, 0.5ng, 1ng, 5ng, 10ng of HSV-1 DNA. The samples were sheared to 500 nt size (ave). A small section of the array is graphed to illustrate the results. Array Oligos that did not hybridize (poorly performing oligos) were marked by black arrows. **B**. The signal box plot for the array samples are shown. Clearly the signal from the standard samples increases with increasing amount of HSV-1 virion DNA. The box plots represent data from the whole genome. The signal for 3 independent 6h PI samples is also shown. **C**. Global profile of microarray experiments by cluster analysis. The columns are samples and the rows are probes. Two groups of microarray samples resolved as major clusters in the heat-map plot. The three infected cell samples (6 hr P.I.) containing viral chromatin clearly segregate from the mock infected (cont.) and virion DNA samples (Std) (which contain no histone but increasing amounts of HSV-1 virion DNA).

A simplified outline of the experimental scheme is shown in Fig. 1. In order to characterize the hybridization efficiency of the oligonucleotides on the array we used dilutions of highly purified HSV-1 virion DNA prepared as described previously [6]. 0.1, 0.5, 1.0, 5.0 or 10.0ng of purified HSV-1 DNA was labeled with Cy3 and mixed with 0.5ug of sonicated cell DNA before being hybridized to the array as described in the Materials and Methods. The hybridization data from a typical section of the genome is shown in Fig. 2A. Clearly some oligos do not hybridize to HSV-1 DNA. We defined an oligo that does not hybridize as a defective, or poorly performing, oligo if the difference of its hybridization signal intensity, measured with 10.0.ng of purified HSV-1 DNA and with the 0.1ng sample, was less than a 2-fold of change. Out of the 2x3045 different tiled oligos covering the HSV-1 genome on the array 344 were defined as poorly performing (5.65%). These defective probes were distributed throughout the genome without any clear clusters. Using our present conditions, we could not detect a signal with 0.1ng HSV-1 virion DNA compared to cell DNA, but we could detect a signal with 0.5ng. Our methodology involves no amplification of the DNA (as occurs in several procedures involving deep sequencing) and thus no errors associated with preferential sequence amplification. In Fig. 2A array oligos are shown to hybridize with different signal strengths to the virion DNA although there should be equal numbers of copies of HSV-1 DNA sequence to hybridize to every oligo. There are separate oligos to hybridize to the terminal and internal repeat genome regions. Although the DNA sequence in the repeats is similar, the oligos are not necessarily the same due to the oligos being ordered as 50mers from the left hand side of the genome. However, when the protected DNA is hybridizing to the arrays, hybridization cannot distinguish between oligos from the “termini” from the “internal” repeats. In order to adjust for the difference in oligo sensitivity, we performed the following calculation to normalize the sample hybridization signals (S_norm_.). First the signal from a cell DNA sample (S_cell_) was subtracted (mock sample). This was considered the background correction. Then the signal was divided by the signal from the 10ng virion DNA sample (S_Vir_.). This was considered the correction for the different array 50 mer oligo hybridization efficiencies. Thus, S_norm_. = (S_raw_-S_cell_)/ (S_Vir-_ S_cell_).

The signal boxplot shown in Fig. 2B confirms that the mock-infected cell nucleosomes have a very small signal over the array, and that the standard virion DNA samples have increasing signal with increasing amount of viral DNA. Furthermore, Fig. 2B shows that the signal from the 6hr PI sample is strong compared to the standard virion samples.

Fig. 2C shows a global profile of all the microarray experiments by cluster analysis (see methods). The 6hr infected cell samples cluster to the left in the heatmap plot, separate from the mock infected and the virion samples, confirming that the array assay can discriminate nucleosome containing viral DNA from non-nucleosome viral DNA.

Our array consisted of 2 sets of overlapping 50mer oligos (overlapping by 25 nucleotides). A data smoothing technique was applied to the array data (see Methods). For example, one 50mer covering 51–100bp on HSV-1 genome (labeled as oligo_75_) and the next overlapping 50mer covering 76–125bp (labeled as Oligo_100_) were combined. Thus there are 3046 combined 50mers (covering 75bp) in the complete HSV-1 genome map. Five positive overlapping 50mers cover 150bp of sequence and were considered the minimum signal to identify a nucleosome (indicated by red balls in nucleosome map figures). Often there did not appear to be a spacer 50bp between 5 positive signals indicating that our array did not have sufficient resolution to identify typical 50bp spacer regions between nucleosomes, or that histone H1 may not always be present between viral nucleosomes, or that the nucleosome position was not precisely fixed with regard to sequence. Because of the number of poorly performing oligos, and other factors which may influence positive signal assignment, four positive overlapping 50mer probes were marked with an orange ball on nucleosome maps and counted as possible nucleosomes (see nucleosome map figures). Our technique will only identify specifically positioned nucleosomes. It will not provide much evidence for a generally dispersed random background distribution—other than an elevated background of hybridization.

### Fraction of DNA MNase Resistant

In order to determine the fraction of HSV-1 DNA present in nucleosomes we digested virally infected tissue culture cells to completion with MNase.

**Fig 3:**
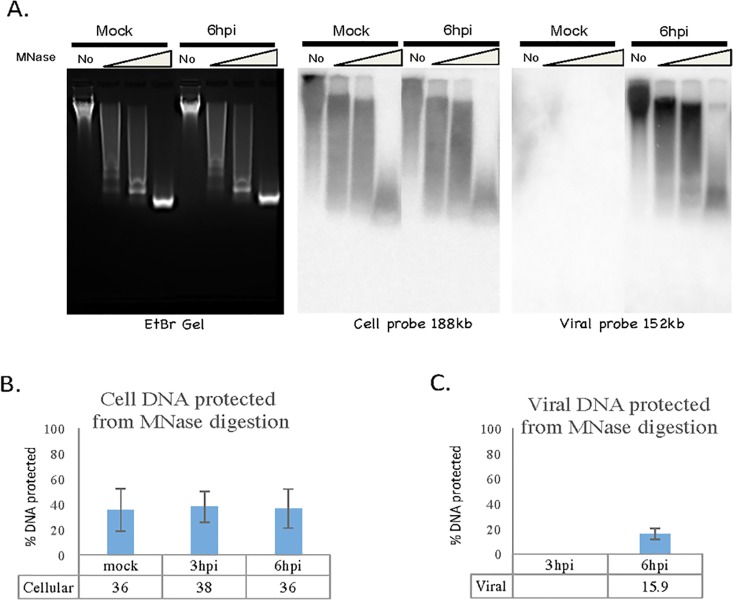
Fraction of DNA MNase Resistant. Similar to our previous nucleosome studies, MNase digestion coupled with gel electrophoresis was used to determine the % DNA protected by nucleosomes [25]. Cells were digested with various concentration of MNase and DNA extracted following which they were separated by 1.5% agarose gel electrophoresis (**A**.), transferred to Hybond—N+ membrane by Southern blot and hybridized to Cell or HSV-1 ^32^P labeled probes (**B. and C**). Total DNA and 150bp protected band DNA were measured by a Typhoon phosphorimager. % DNA protected was calculated as 150bp DNA band divided by total DNA. All data are the averages of three independent experiments, and error bars represent the standard deviation.

Sy5y cells were infected with HSV-1 strain F at a MOI of 5 and harvested at 0, 3, and 6, P.I. Harvested cells were digested with various concentration of MNase and DNA extracted following which they were separated by 1.5% agarose gel electrophoresis, transferred to Hybond—N+ membrane by Southern blot and hybridized to cell or HSV-1 specific ^32^P labeled DNA probes as described in the Materials and Methods (Fig. 3A). For cell DNA the probe was a 188kb bacmid sequence (clone RP11-34L24 from RPCl human BAC library 11). The 3hpi gel data is not shown as the level of viral DNA was at the limit of detection. Approximately 36% of cell DNA was found nuclease resistant (Fig. 3B), as determined by phosphorimage scanning of 150bp density on the southern blot filters, as shown in Fig. 3A. Data from MNase-seq experiments on the human genome suggest 50% resistance [28]. For HSV-1 DNA detection the probe consisted of a mixture of 5 cosmid clones covering the entire 152kb viral genome [29]. At 6 h post infection the fraction of HSV DNA resistant to infection was 16% (Fig. 3C). No value could be determined for 3hpi due to the low hybridization signal, which may be due to technical limitations of the assay. Thus HSV-1 DNA appears to contain approximately half as much MNase resistant DNA as cell DNA.

### Nucleosome distribution on Immediate Early genes at 6hr PI

**Fig 4:**
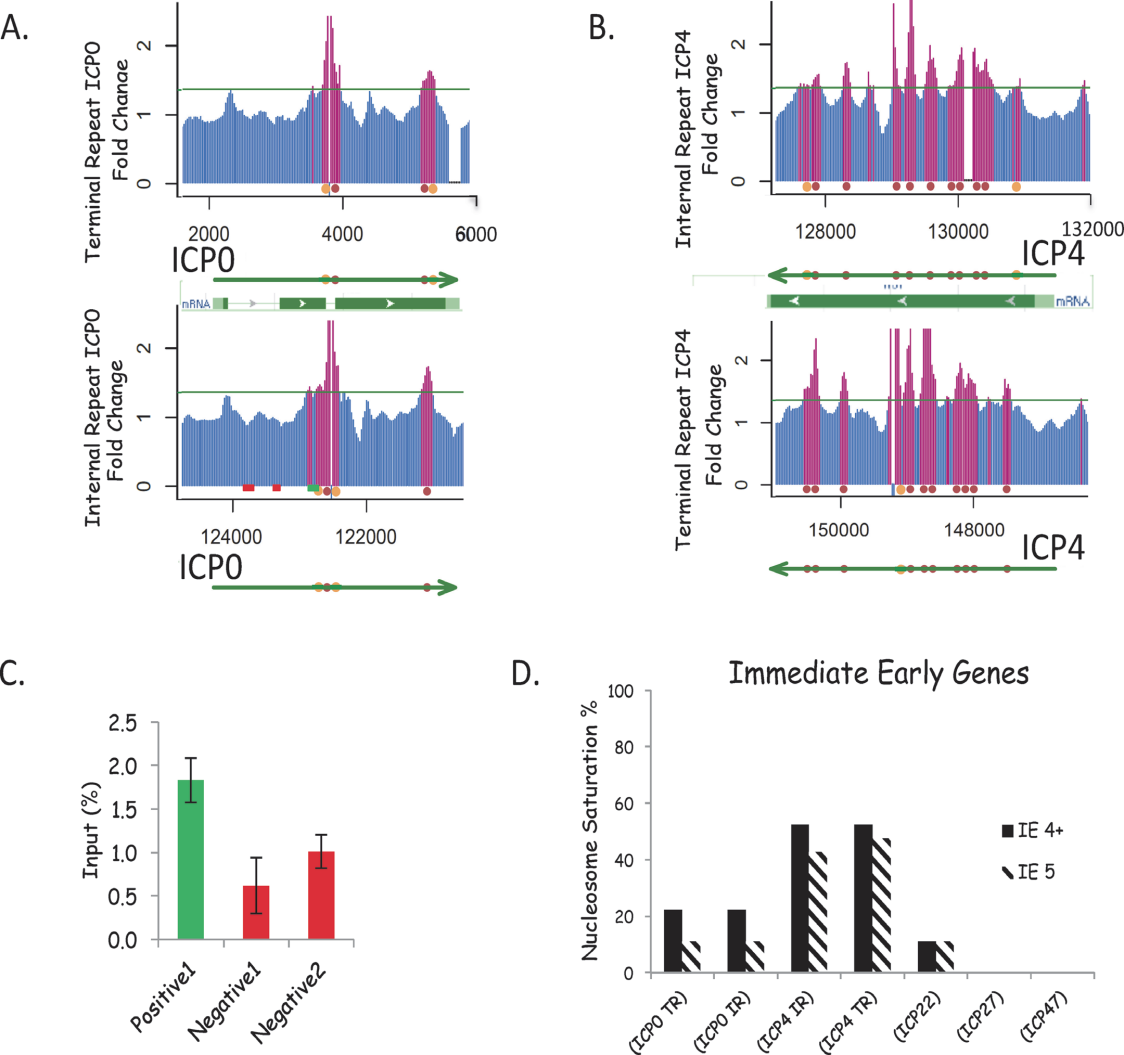
Nucleosomes on Immediate Early genes. **A** and **B**. The nucleosome signal is shown for the region covering two immediate early genes, which are diploid in the HSV-1 genome. The two gene copies have been inverted and aligned with each other to show that the array signal is similar but not identical. Spaces in the graph lines are “poorly performing” oligos where no signal could be measured. Between the array signal information we have mapped the transcribed gene (green). The red balls indicate the predicted position of nucleosomes with 5 consecutive positive hybridizing oligos. The orange balls indicate where we have inferred a nucleosome from 4 positive oligos and assumed there is a fifth positive oligo that is not recorded because of a “poorly performing” oligo, or some other technical problem. Gene features from the NCIB database are also shown. **C** To confirm our nucleosome positioning we used ChIP and Q-PCR (see Methods), selecting primers to amplify regions of ICP0 that we designated as having a probable nucleosome (green), or NFR (red). **D**. Nucleosome Saturation for each Immediate Early gene. The predicted number of nucleosomes for each IE gene were compared to the maximum number of nucleosomes possible (assuming a standard cell 200nt/nucleosome repeat pattern) to give the nucleosome saturation, which is expressed as a %.

The HSV-1 replication cycle in tissue culture cells is comparatively fast and new virions are produced in about 18h. During this time viral gene expression takes place in a well organized, temporally ordered cascade of transcripts [30,31]. The immediate early genes are synthesized at their highest rates by about 3–4hr P.I., the early genes peak at about 5–7hr and the late genes increase in level until at least 12h P.I. Thus we chose 6h P.I. as a time for our initial study of nucleosome positioning—a time P.I. at which we had previously shown there is a maximum amount of nucleosome protected HSV-1 DNA [7]. We first examined the hybridization signal to the IE genes. Fig. 4 shows the array hybridization data for ICP0 (a) and ICP4 (b). The terminal and internal copy of the genome are aligned in the same direction, with a diagram of the transcriptional elements and translational open reading frame mapped (NCBI). Nucleosomes are designated by red or orange balls on the green arrow, which designates the transcript. Red balls represent nucleosomes designated by 5 consecutive positive signals, orange by any 4 positive out of 5 consecutive signals. Blue bars in the plots denote the hybridization signal to oligomers on the array below the cut-off level, and red bars hybridization above the cut-off level. The green line marks the cut-off level for a positive nucleosome signal (calculated as described above).

Clearly the patterns of hybridization for the two copies of the ICP0 gene are similar, but not identical (Fig. 4A). Because the oligos are 50nt sequences, measured from the left hand end of the prototype genome sequence, the sequence of the individual oligos from similar positions on the two genome copies of the ICP0 gene (terminal and internal repeat regions of the genome) do not necessarily match. However there is clearly a strong similarity in the pattern of hybridization. This is also seen for the other diploid IE gene ICP4 (Fig. 3B). In this case the pattern, although similar appears somewhat less so. Whether this is due to variance in our data, or differences in the unique sequence genome locations surrounding the terminal and internal repeat elements, is not known.

In order to confirm our nucleosome positioning we used chromatin immunoprecipitation (ChIP) assay with qPCR, selecting primers on ICP0 gene that we designated as having a probable nucleosome (green), or NFR (red). The PCR signal shown in Fig. 4C confirms the hybridization strengths seen on our arrays and thus nucleosome mapping for ICP0.

For the ICP0 gene it is striking how few nucleosomes there are at 6h PI. Only 11–22% of the possible nucleosome sites are occupied, depending on whether one scores 5, or 4 out of 5, consecutive positive signals as a nucleosome (Fig. 4D). The presence of nucleosome free regions (NFRs), a feature of actively transcribed cellular genes, is seen for ICP0 and also obvious over most other HSV-1 genes [32,33,34]. The presence of poly(dA:dT) regions has been suggested as a factor favoring NFRs [35,36]. However, (dA:dT) regions do not occur in the ICP0 gene due to its high GC content (69%). Possibly the NFRs are due to some other influence such as active transcription. The presence of NFR over promoters of HSV-1, such as ICP0 and ICP 4 at 6hrPI, may also be due to active transcription, since it is unlikely that nucleosomes and RNA polymerase complexes can occupy the same sequence of DNA [37]. Interestingly, Herrera and Triezenberg showed, by ChIP experiments, that at 2h P.I. H3 histone was underrepresented on IE promoters [14].

The analysis of nucleosome saturation for all 5 of the IE genes is shown in Fig. 4D.

In order to determine the saturation of nucleosomes on the viral genes we made the following calculation. Assuming a nucleosome occupies 200 nt of DNA sequence (150bp MNase protected +50 bp spacer), the theoretical maximum occupancy on each gene, was calculated by dividing the transcribed gene size (RNA start nt to poly AATAAA signal sequence) by 200. It does not take into account the promoter region or the 3’ termination region past the poly A processing site. The % saturation was calculated as the measured nucleosome number divided by the calculated maximum number of nucleosomes. To account for occasional “poorly performing” oligos and other factors lowering our assignment of nucleosomes we included 4+ positive signals as nucleosomes, designating them by orange rather than red balls. An average of 18% saturation was seen for IE genes at 6hr PI (23% if including 4+ assignments). Interestingly ICP0 had lower saturation (22% 4 out of 5 and 11% 5 positive) than ICP4, which showed 52% for 4 out of 5 and 43% for 5 positive on the IR copy and 48% for 4 out of 5, or 5 positive, on the terminal repeat gene. Both the gene for ICP 27 and 47 had no nucleosomes, suggesting that they are not essential for IE gene expression.

### Nucleosomes distribution on Early Genes 6hr PI is similar to that of IE genes

**Fig 5:**
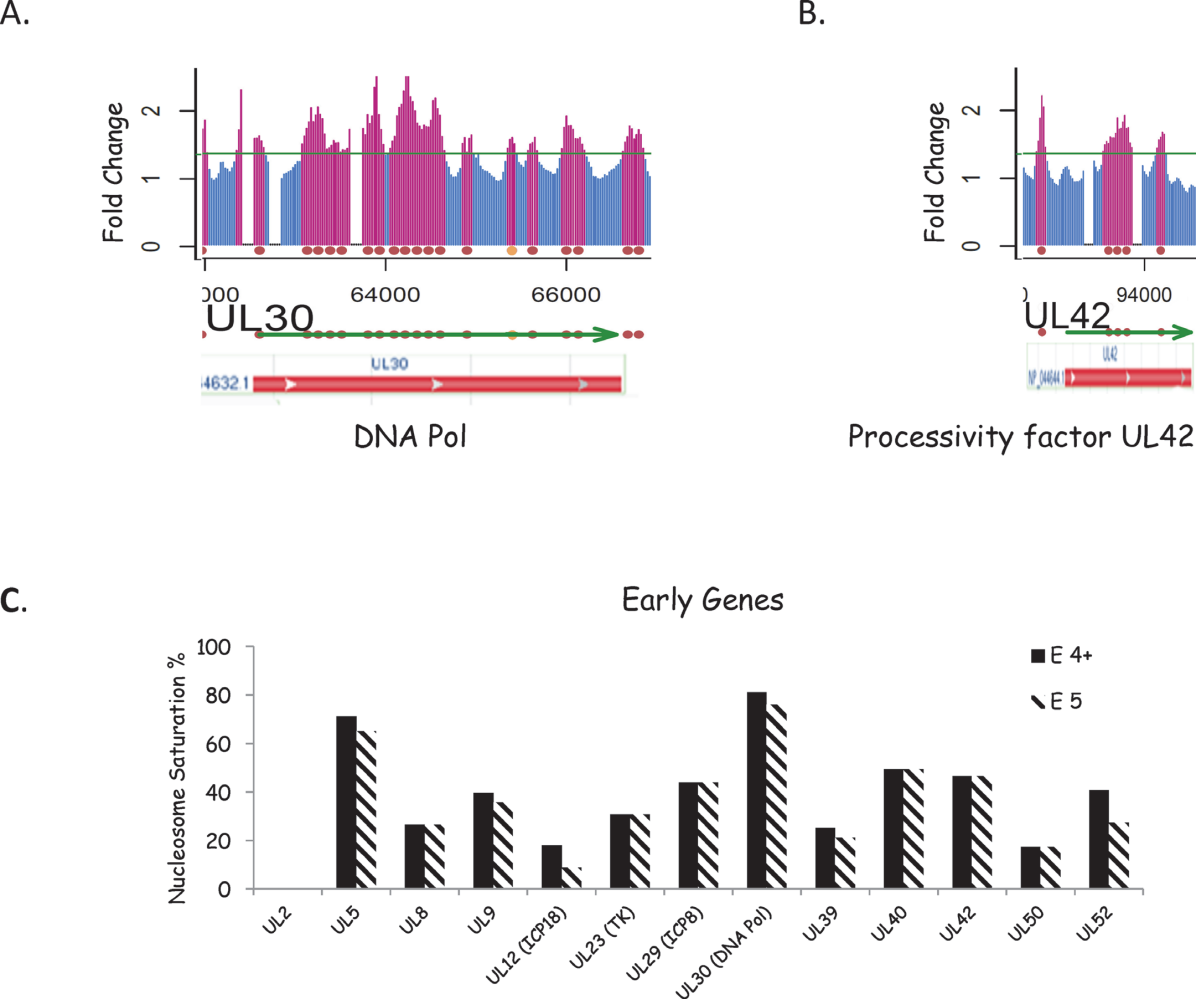
Nucleosomes on Early genes. **A. and B** The nucleosome signal is shown in the region covering the gene for DNA polymerase (UL30) and its processivity factor (UL41). There is one copy of these genes located in the unique long segment of the viral gene. Below the array signal information we have mapped the transcribed gene and the red or orange balls indicate the predicted position of nucleosomes. Gene features from the NCIB database are also shown. **C**. Nucleosome Saturation for each E gene. The predicted nucleosomes for each E gene were compared to the maximum number of nucleosomes (assuming a 200nt/nucleosome repeat pattern) to give the nucleosome saturation, which is expressed as a %.

At 6h P.I. gene expression of the early genes should be peaking [30]. The nucleosome saturation on early genes at 6hr PI on average was higher than that on IE genes; 38% (4+) and 34% (5 positive). Although there was a trend there was not a statistical difference by 2-tailed T-Test or non-parametric Wilcoxon rank sum test. Fig. 5A and B shows the nucleosome pattern for the DNA polymerase and processivity factor genes (U_L_30 and U_L_42). The nucleosome density was higher than that seen on the IE genes, and the nucleosomes tend to be clustered on the genes. There were 81% (4+) and 76% (5 positive) saturation for the polymerase gene, and 47% saturation for the processivity gene whether calculated with 4+ or 5 consecutive positive signals. Fig. 5C shows the nucleosome saturation for all thirteen early genes. Interestingly UL 30 (DNA pol) had the highest level of nucleosome saturation. It is not clear if the trend to higher nucleosome saturation on the early genes than the IE genes at this time is because they are being more actively transcribed, or because they have lower GC content, or for some other reason. One early gene, UL2 had no nucleosomes like the ICP27 IE gene, again suggesting that they are not necessary for individual viral gene expression.

### Nucleosomes distribution on Late Genes at 6hr PI is similar to Early genes

**Fig 6:**
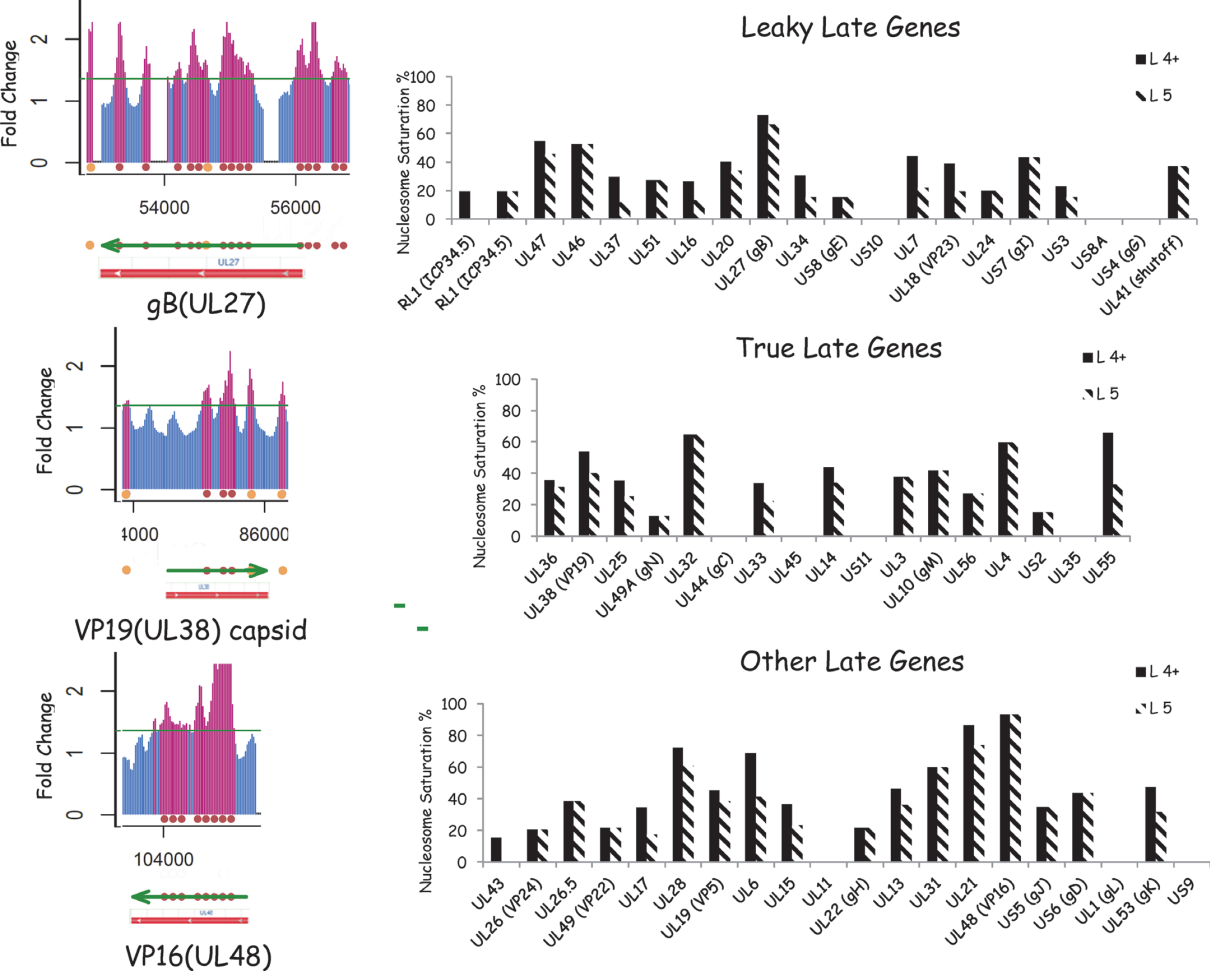
Nucleosome on Late Genes. **A**. The nucleosome signal is shown in the region covering the late gene VP16 (UL48) a tegument protein and transactivating factor for transcription of IE genes, VP19 (UL38) a capsid protein, and gB (UL27) a glycoprotein found in the viral membrane. Spaces in the graph lines are “poorly performing” oligos. Below the array signal information we have mapped the transcribed gene and the orange balls indicate the predicted position of nucleosomes. Gene features from the NCIB database are also shown. **B**. Nucleosome Saturation for select L genes. The predicted nucleosomes for each L gene were compared to the maximum number of nucleosomes (assuming a 200nt/nucleosome repeat pattern) to give the nucleosome saturation, which is expressed as a %. Late genes were sub-grouped into true late genes (which are not expressed prior to DNA replication; B1) leaky late genes (which are expressed at a low level prior to DNA replication; B.2) and Late genes (which were not sub-characterized; B3).

**Fig 7:**
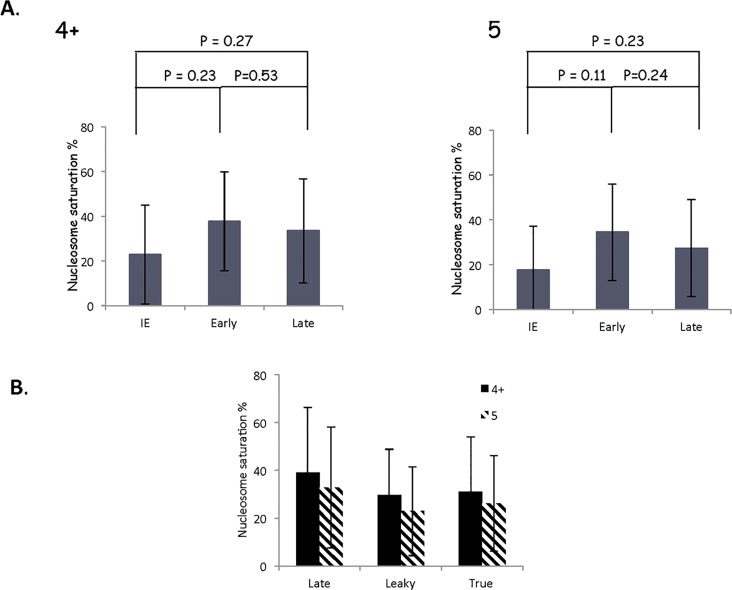
The nucleosome saturation on IE, E, and late genes is statistically similar. **A**. The predicted nucleosomes for each gene kinetic class (IE, E, L), and **B**. sub-class (late, leaky, true) were compared to the maximum number of nucleosomes to give the nucleosome saturation, which is expressed as a %. The average % saturation of IE genes was not significantly different than the average saturation of the E genes or that of the Late genes (57 of 66 analyzed) using a non-parametric Wilcoxon rank sum test, although the IE genes did trend lower. Error bars represents the standard deviation.

At 6h PI, although the L genes are being expressed they have not yet peaked [30]. There are about 66 genes characterized as transcriptionally “Late” genes in the HSV-1 genome. Of these we have characterized about 57. Fig. 6 shows UL27 (gB, a virion membrane protein), UL38 (VP38, a capsid protein), and UL48 (VP16, a transcription factor and tegument protein). The average nucleosome saturation of late genes was 33% (4+) and 27% (5 positive)—which was not significantly different from the Immediate Early or the Early genes (Fig. 7). There are several late genes that appear to have no nucleosomes covering them (US8a, US4, US50, UL44, US11, UL55, UL11, UL1). Some are short genes; however, the fact that no nucleosomes are present supports the hypothesis that nucleosomes are not essential for individual viral gene expression. Some late genes have very high numbers of nucleosomes (UL56, UL 32, UL26.5, and UL6). Why these genes, expressed late in the infectious cycle, should have so many stable nucleosomes covering them at 6h P.I. is unclear. A high level of nucleosome saturation is not confined to Late genes—the saturation over an early gene is 80% (UL30; Fig. 6). Thus high levels of nucleosomes are unlikely to be due to the later onset of transcription for the “Late” genes. This is further supported by the fact that genes classified as “true late” genes, which are only expressed after DNA replication, have similar levels of saturation as leaky late” genes, expressed in small amounts prior to DNA replication (Fig. 6 and Fig. 7). Although the nucleosome saturation of the IE genes trends lower than the E or L genes, this difference is not statistically significant by the non-parametric Wilcoxon rank sum test. (Fig. 7). Late genes which could not be classified to the true or leaky late class (from the literature) were presented in class by themselves (Late).

### The LAT gene nucleosome saturation is similar to an IE gene

**Fig 8:**
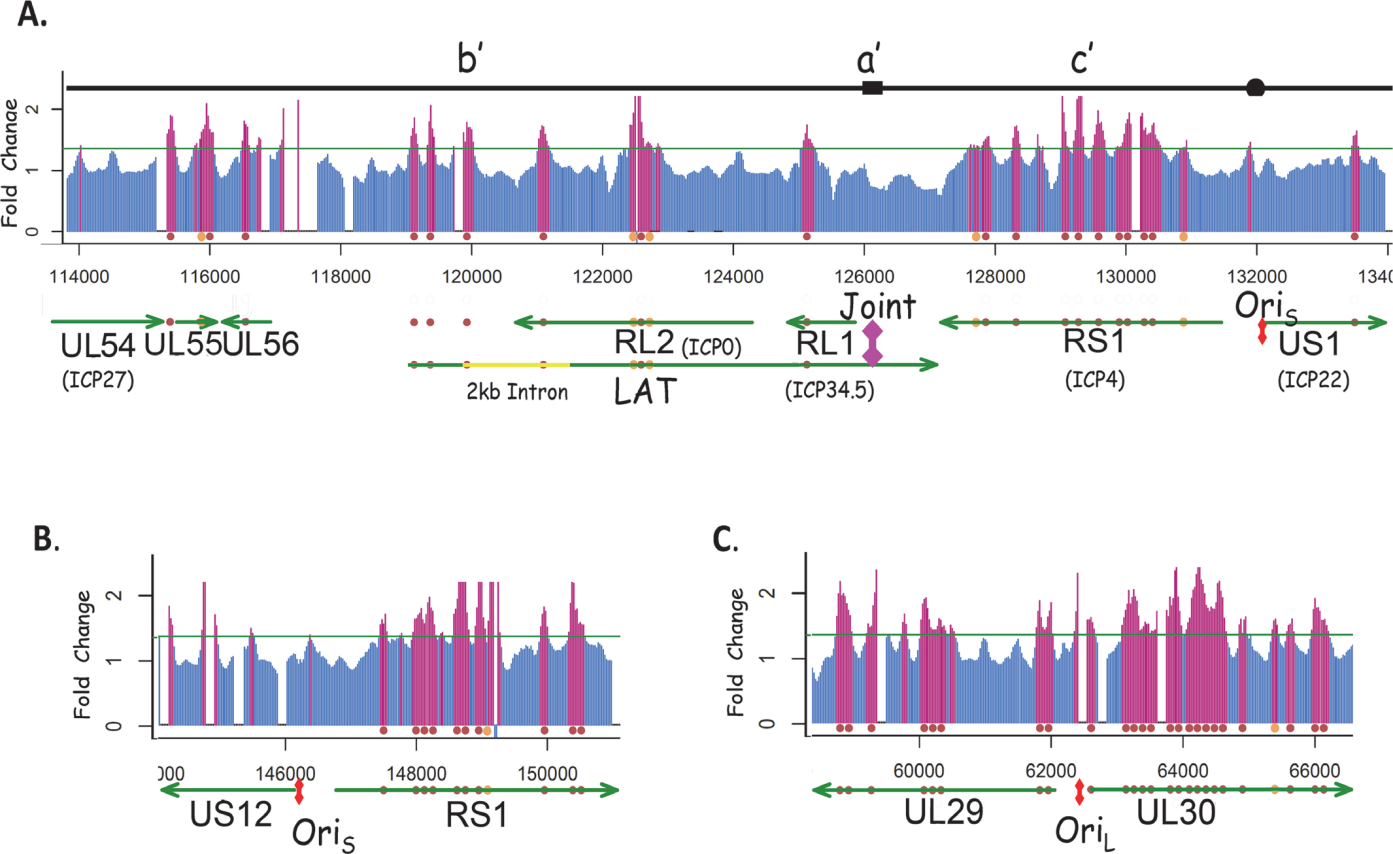
The “joint” and “Ori” regions are low in nucleosomes. **A**. The black line maps the b’, a’ and c’ repeat regions of the viral genome surrounding the “Joint” of the Long and Short segments. The hybridization signal to array oligos is shown as blue lines below and red for above our fold change cut off. Spaces in the blue and red lines are “poorly performing” oligos. Transcripts are mapped below as green arrows, with nucleosomes presented as red or orange balls. At 6h PI the region of the genome including the “joint”—where the long and short segments of the HSV-1 genome isomerize—is not coated with nucleosomes. (**A**.). ORI_S_ has no nucleosomes near it (**A**. and **C**.) whereas ORI_L_ has some. **B**.

Transcripts from the latency associated transcript (LAT) gene are the major viral gene products detected in latently infected tissue [38,39,40]. However, the LAT gene is also transcribed during lytic infection [41,42] and thus we have included its analysis in this study (Fig. 8A). The LAT 2kb stable intron accumulates late in infected tissue culture cells but can be detected in small amounts at early times[41]. The nucleosome saturation of LAT is 19% (4 out of 5) and 14% (5 positive). Thus it looks like an IE gene in terms of nucleosome saturation at 6hr P.I.

Nucleosome accumulation appears to favor the 5’ end of the gene. However it is not clear if this reflects the fact that the “Joint” region of the genome overlaps with the 3’ end of the LAT gene and this region lacks nucleosomes. Interestingly the Late gene, ICP34.5, also overlaps the LAT gene 3’ end and has only one nucleosome covering it (19% saturation). It is possible that this is due to the proximity of the “Joint” region dominating the chromatin structure over ICP34.5 and the LAT 3’ gene regions (Fig. 8A).

### The Joint and Origins of DNA replication are devoid of nucleosomes

The DNA sequence of the HSV-1 genome is composed of two unique DNA segments bounded by repeat elements and the genome is capable of existing in 4 equivalent isomeric forms [43]. Although the HSV-1 genome is a linear molecule in the virion, the genome is circularized on entering the infected cell nucleus [44,45]. Thus there are 2 copies of the joint region of the genome in infected cells. These regions contain signals for DNA packaging into the viral capsids, and the elements that allow genome isomerization. The data show that the joint region does not appear to have any nucleosomes covering it at 6h P.I. (Fig. 8A).

HSV-1 has 3 origins of DNA replication. The diploid origin in the Repeat Short element of the genome (Ori_S_) is not bounded by nucleosomes (Fig. 8B). Whereas the origin in the Unique Long region of the viral genome (Ori_L_) is bounded by nucleosomes (Fig. 8C). The two Ori_S_ are flanked by two IE genes, (ICP4 and ICP22; and ICP4 and ICP47). Ori_L_ is flanked by two E genes, DNA polymerase and ICP8 (ss DNA binding protein). It is not clear whether the presence of IE promoters flanking the Ori_S_ contribute to the lack of nucleosomes flanking the Ori_S_, while the presence of two early promoters flanking the Ori_L_ contribute to the nucleosome close to Ori_L_. It is interesting that a nucleosome at Ori_L_ appears to be a “phasing” nucleosome at the start of the UL 30 (DNA pol) gene, and UL 30 has one of the highest saturations of nucleosomes.

### Relationship between GC% and Nucleosome saturation

**Fig 9:**
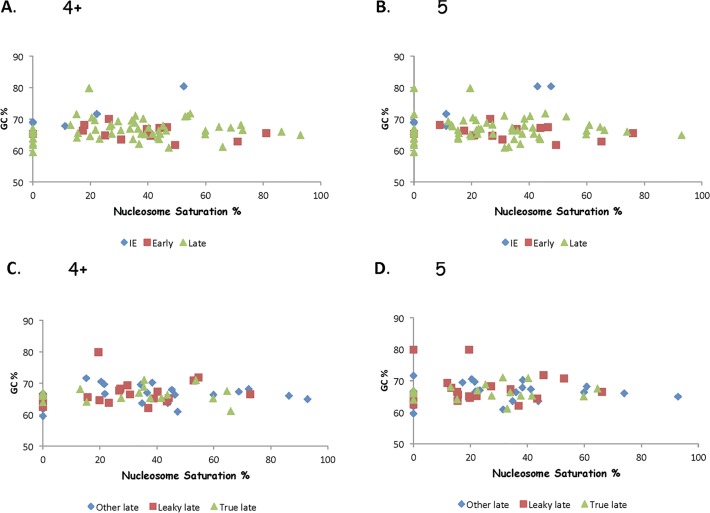
GC content does not predict Nucleosome Saturation. **A**. and **B**. Nucleosome Saturation and GC content for Immediate Early, Early, and Late genes. **C**. and **D**. Nucleosome Saturation and GC content for Late, Leaky Late, and True Late genes.

Genome wide nucleosome studies suggest that DNA sequence is a major determinant of nucleosome positioning, however the sequence elements involved remain obscure despite many years of study [46,47,48]. It has long been thought that AT favored and GC disfavored nucleosome occupancy on cell genes [49]. Thus it is possible that the high GC content of the HSV-1 genome is inhibiting nucleosome deposition based on this sequence bias. However, Table 1 shows that there is a rough inverse relationship between GC content of HSV-1 genome segments and nucleosome density. This is not supported by comparison of the GC% to the nucleosome saturation for individual genes in the 3 gene classes. Fig. 9 (A and B) reveals a slight correlation between GC content and nucleosome saturation for IE genes, but not for E or L genes. However, examination of GC% and nucleosome saturation for genes classified as late, leaky late, or true late reveal no correlation (Fig. 9 C and D). Thus, because GC content is higher in DNA sequence encoding IE genes than in DNA sequence encoding other genes of the HSV-1 genome, it is likely that other factors are influencing nucleosome positioning—for example the level of gene expression or the level of CpG.

**Table 1:** Comparison of nucleosome distribution and GC content of regions of the HSV genome.

	**TR**_**L**_	**U**_**L**_	**IR**_**L**_	**IR**_**S**_	**U**_**S**_	**TR**_**S**_
Max # of nucleosome	46	540	46	33	65	33
Actual # of nucleosome (4+)	9	222	8	11	13	11
Nucleosome Saturation % (4+)	20	41	17	33	20	33
Actual # of nucleosome (5)	6	185	6	9	12	10
Nucleosome Saturation % (5)	13	34	13	27	18	30
GC %	72	67	72	80	64	80

### Relationship between Introns and Nucleosome saturation

Some studies have shown that nucleosomes are preferentially placed at exons in cell genes [50]. There are no nucleosomes over the 1^st^ intron and 1–2 nucleosomes over the 2^nd^ intron, out of a total of 2–4 nucleosomes mapped over the entire ICP0 gene (Fig. 4A). This would argue for an intron preference for positioning of nucleosomes on the ICP0 gene—unlike cell genes—given that there is much more exon than intron sequence in the gene. However because the LAT gene overlaps the ICP0 gene it is possible that it also influences nucleosome positioning in this region. However there are too few genes in HSV-1 that have introns to make any hard conclusions about this point.

On the other hand, the 2kb stable intron of the LAT gene has a nucleosome saturation of 20%- higher than that of the whole LAT gene 14% to 19% (5 positive—4+ positive), arguing that in the case of the LAT gene chromatin structure is dictated by a different mechanism from that governing deposition on cell gene DNA (Fig. 8A).

## Discussion

The advent of high-density arrays has led to the possibility of mapping nucleosomes over wide regions of genomes, and the use of chromatin immunoprecipitation to select specifically modified histones, has further developed the field by allowing the mapping of nucleosomes containing modified histones. Even more recently the use of deep sequencing technologies has provided an alternative to the array hybridization technology through direct sequencing of short DNA pieces. Although little work mapping nucleosomes has been reported for HSV-1, recently Gunther and Grundhoff published data on the epigenetic landscape of the latent Kaposi Sarcoma-associated Herpesvirus genome [51]. In the present study we have used array hybridization technology to map the positions of nucleosomes on the HSV-1 genome at 6 h. post infection. This time was chosen as a time when viral gene expression is well established, viral DNA replication is starting, and nucleosome deposition on the HSV-1 genome is near maximum. It is expected that we are examining a mixture of replicating, transcribing and input viral genomes—but not virion DNA as this is protected from MNase digestion.

It has been observed that during lytic infections, HSV-1 DNA can form complexes with the properties of unstable nucleosomes [52,53]. It is possible that these are non-nucleosomal histone-DNA intermediates as described by Kadonga et al., [54]. Because we are not fixing our chromatin prior to MNase treatment it is unlikely that we are observing these unstable nucleosome intermediates. Thus our results reflect the positioning of tightly bound nucleosomes. Data from the complete MNase digestion of cell and viral DNA (Fig. 3) show that there is approximately half as much viral DNA protected from MNase digestion as cell DNA, suggesting that the density of nucleosomes on viral DNA is half that of cell DNA at 6h post infection in tissue culture cells.

Furthermore, because we are selecting 150bp DNA fragments following gel electrophoresis, it is likely that we are not selecting DNA fragments protected by partial nucleosomes, transcription factors, or other DNA binding proteins.

### Control samples (virion DNA) show that most of the array 50mers can give a signal

The development of high-density oligonucleotide arrays has led to the development of genome wide studies of nucleosome positioning. Our array consists of overlapping 50mers covering the entire HSV-1 genome. There are two problems with the data gained from hybridization of samples to this array. First, cellular nucleosome 150bp protected DNA fragments, which constitute the majority of the sample, may hybridize to array oligomers to give a background signal. Thus we prepared and hybridized samples from uninfected cells (mock infected, negative control). This signal was subtracted from HSV-1 infected samples. A second problem with the array is that each oligomer hybridizes with a different efficiency—and some oligomers do not hybridize at all. To compensate for the differences in hybridization efficiency of the each oligomer, the array was hybridized with HSV-1 virion DNA (which should contain 1 copy of each oligomer sequence). There were 344 (5.65%) of our 2x3046 x tiled 50mers that were classified as non-hybridizing (poorly performing) oligomers. Mapping was not possible at the genome position of these oligomers, however their distribution appeared random throughout the viral genome and thus their impact on the data is minimal (see S1 Fig.).

### Histone deposition is not random

The data from 3 independently prepared samples at 6hr post infection show that the distribution of nucleosomes on the HSV-1 genome although disorganized, as described by Placek and Berger [16] is not random and that there are distinct positions or regions of the HSV-1 genes favored by nucleosomes (Figs 4,5,6,8). This may be due to insufficient time for chromatin remodeling (an ATP dependent process which would evenly space the nucleosomes [13]) to occur during the viral lytic cycle and has also seen for nucleosomes on the CMV genome [55]. Our technique will not reveal a random background of nucleosomes if present. A random distribution would be expected to produce an average signal all over the genome yet we do see nucleosome free regions (NFR). Interestingly, where there are regions of genes covered with many contiguous nucleosomes (mainly early and late genes) there do not appear to be well defined spacer regions (~50bp) where H1 histone would be bound. This supports a “fuzzy” rather than “phased” model of nucleosome positioning [48] and suggests that though there are specific regions where nucleosomes are found, the exact position of a nucleosome on the HSV-1 genome may vary within a population of genomes. Also, the differences in hybridization signal strength, even after correction for 50mer hybridization efficiency, suggests that nucleosomes do not occupy all genome sites with the same frequency in a population of genomes.

It is possible that a viral protein is bound to some of the HSV-1 genomes altering the gap between close nucleosomes, as in the case of the hepatitis B virus minichromosome—which was shown to be unusually short in nucleosomal spacing (10%) due to the presence of a core viral protein [56]. There is no direct evidence of histone H1 binding between nucleosomes on HSV-1 DNA during lytic infection.

### Nucleosomes are located at preferred positions on the viral genome

From published studies it is clear that promoter regions of actively transcribed cell genes are generally nucleosome free, as are the 3’ termini of transcribed cellular genes (reviewed in [48]). In HSV-1 infections at 6hr PI, a peak time of viral gene expression, there are not only NFRs over promoters and 3’ regions but also NFRs in internal regions, as shown in Fig. 4A and B, Fig. 5A and B, and Fig. 6. Most promoters are nucleosome free, and in fact we find all of the immediate early promoters are nucleosome free (see data S1, S2 Figs and primary). This may be a consequence of transcription factors such as VP16 [14,57]. Five of 13 (38%) early promoter regions have a nucleosome and 21/57 (37%) late promoter regions have a nucleosome. On average a +1 nucleotide position nucleosome occurs in 40% of the HSV-1 genes at 6hr PI. This nucleosome, which is believed to phase the following nucleosomes on cell genes clearly does not phase nucleosomes, or phases nucleosomes only to a limited extent on HSV-1 genes because there are gaps (NFR) within the genes. One need look no further than gB (UL27) to see a nucleosome at the 5’ end of the gene and NFR within the gene (Fig. 6). Clearly the first nucleosome is not positioning the remainder of the nucleosomes. What causes the gaps in our nucleosome map? Some may be artificial, caused by poorly performing oligos. For example the gap around nucleotide position 54,000 in gB (UL27) may be due to poorly performing oligos within that region (Fig. 6).

The large NFRs in the transcribed regions of the ICP0 (Fig. 4A) might suggest that because this gene is highly expressed there is not sufficient time to allow for nucleosomes to form on it. Furthermore because chromatin remodeling requires ATP and time, the lack of order in the nucleosomes, as seen in the lack of a ladder on partial MNase digestion, may be due to insufficient time for remodeling to occur [13].The fact that the latent genome shows an ordered nucleosome pattern would support this argument [58].

There are some positions that are not occupied, or are occupied at a frequency below the detection level. However, it is possible that these positions may be occupied at other times of infection, or in other types of infected cells. It is also possible that there is a background of randomly positioned nucleosomes on the population of viral genomes in our sample, which may account for the general high level of background signal seen on our array. Lastly, it is possible that some of the NFR were occupied by loosely bound or labile nucleosomes, such as those described by Lacasse and Schang (52).

It is interesting to note that nucleosomes tend to be clustered in the middle of genes with the ends free (see S1, S2 Figs and primary data). Also, that short genes tend not to have nucleosomes covering them (for example UL45, gG, US8a). It is tempting to think that transcriptional machinery occupies the ends of the genes inhibiting nucleosome deposition, thus short genes have no free space for nucleosomes in their internal sequences. However, some short genes do have an occasional nucleosome (UL51, UL 55, UL56).

Besides being located within transcribed genes NFRs or nucleosome depleted regions are located at the origins of DNA replication (Ori_L_ and Ori_S_). The Joint region of the genome also appears to be nucleosome free. As this region is neither the binding site of RNA polymerase or DNA polymerase the reason for its lack of nucleosomes is unclear. It was previously noted that nucleosomes may fall off the ends of linear DNA molecules resulting in a random pattern of nucleosomes [59]. However at 6hr post infection the genome is thought to be endless or circular [8,44].

### Diploid genes, in repeat elements of the genome, give similar patterns

The observation that the 2 copies of ICP0, and those of ICP4, give similar patterns of nucleosomes gives us confidence that our array is functioning in a reproducible manner. Although the pattern is similar it is not identical. This may be due to the fact that the 2 regions encoding the diploid genes are not necessarily covered by the same 50mers on the array (due to the 50mers being positioned from the 1^st^ nucleotide in the genome and the diploid copies of the ICP0 and 4 gene not occurring at a multiple of 50 nucleotides apart), thus affecting the efficiency of hybridization. We do not think we are differentiating the hybridization between the repeat regions.

### IE genes tend to be more sparsely covered with nucleosomes, compared to genes of other kinetic classes

One of the interesting findings is the trend to lower nucleosome density on the IE compared to other classes of genes with respect to nucleosome density or saturation. Because the number of IE genes is small it limits the statistical power available to detect the true difference in saturation, however we do see a trend. The IE genes, with about 0–50% of the maximum possible number of nucleosomes covering their DNA, have fewer nucleosomes on average than seen on the E genes (Fig. 7). The reason for this is not clear. Is it due to their earlier start in the viral transcription cascade or is it due to the higher GC content of the genome in the repeat elements of the viral genome (where IE genes are encoded)?

The HSV-1 genome is high in GC content (69%) compared to cellular genomes (40%) in which a relationship between GC content and nucleosome positioning has been identified [60,61]. CpG methylation has also been related to nucleosome positioning both positively and negatively [62]. However CpG methylation is unlikely to account for the trend to a difference in nucleosome occupancy on the E and L genes compared to the IE HSV-1 genes as no methylation has been found on HSV DNA [63,64].

Some genes have no stable nucleosomes suggesting that stable nucleosomes are not essential for individual gene expression. Members of all 3 kinetic classes of HSV-1 genes show this property. All are short in length, suggesting the presence of transcriptional complexes and other related proteins at the 5’ and 3’ end of viral genes inhibit nucleosome deposition—longer viral genes have space, and thus more time between each transcriptional event, for nucleosome deposition (assuming equal rates of transcription). Alternatively it is possible that these genes are only covered by unstable (fluid) nucleosomes[53].

### Which factors dictate nucleosome occupancy?

From yeast genome studies on nucleosome positioning with respect to DNA sequence there are two hypothesis governing positioning. One suggests positioning based on the genomic code [65], the other suggests positioning based on site occupancy—statistical positioning [66,67,68]. CMV genomes during lytic infection have nucleosomes bound in a non-random and predictable manner [55]. During early infection the nucleosomes appear to associate with viral DNA according to their intrinsic DNA sequence preferences. Later in the infectious cycle the nucleosomes positioning is mostly determined by non genetic factors. The generation of EBV nucleosome maps has also been performed and combined with high throughput sequencing datasets for human lymphoblastoid cell lines to generate interactions between the EBV genome and host factors.[69]. Nucleosomes were shown to strongly occupy the transcriptionally silent Cp promoter in the type 1 Burkitt lymphoma cell lines (expressing the EBNA2 family of genes), but did not occupy the transcriptionally active Cp in type 111 lymphoblastoid cell lines (expressing EBNA2 family genes)[70]. The genomic code-positioning hypothesis argues that the genome determines the organization of nucleosomes via intrinsic DNA sequence preferences, while the nucleosome occupancy hypothesis argues that the intrinsic DNA sequence preferences are but one factor that influences in vivo nucleosome organization, and there are other factors influencing positioning that differ from the “genetic code”. Nevertheless, both groups agree that nucleosome sequence preferences can explain nucleosome positioning to some extent. One of the intrinsic DNA sequence elements that may influence positioning is dinucleotide periodicity. This influence is found in various organisms including yeast, worm, fly, chicken and human [65]. Poly(dA:dT) has been shown to disfavor nucleosome formation in vitro [71]. Furthermore, yeast promoters are AT-rich and nucleosome-depleted [72], and higher GC% DNA regions have been correlated with more nucleosome [73]. These findings are somewhat supported by our data from 6h P.I. HSV-1 DNA, where we clearly see few nucleosomes round viral promoters (Figs. 4, 5, 6, and 8). However, there is a lack of nucleosomes in the GC rich IE gene regions compared to the less GC rich E and L gene regions (Fig. 9A and B). Nevertheless, it should be remembered that the whole HSV-1 genome is about 69%GC compared to the human genome (about 40%) thus the HSV-1 genome is not representative of typical cellular DNA. Interestingly, cell ribosomal DNA has a high GC content and has an unusual nucleosomal pattern as revealed by MNase digestion [74].

From Fig. 9A and B it can be seen that although some IE genes are more GC rich than other genes in the HSV-1 genome (avg. 68% GC), the GC content of some E and L genes overlaps with those of the IE genes. Early gene ICP 18 has a GC content of 68% similar to IE gene ICP22, and the saturation of ICP18 is 18/9% similar to 11/11% (4+/5positive) for ICP22. However L gene UL26 (VP24) has a GC content of 70%, similar to ICP27 or ICP47, yet it has a higher nucleosome saturation of 20% compared to 0% for ICP27 and ICP47. Thus HSV-1 genes with similar GC content can have markedly different nucleosome saturation levels. Nevertheless, in general IE genes have higher GC content and lower nucleosome saturation levels than E or L genes.

### Nucleosomes prefer clustering towards the 5’ region of most early and late genes

Array analysis of cellular genomes have shown that nucleosomes are positioned around the beginning of genes in a similar way, with a NFR flanked by two well positioned nucleosomes often referred to as the +1 and -1 nucleosome [75]. Interestingly, although there is a NFR over most of the viral promoters there are not well-defined +1 (phasing) nucleosomes at the start of most viral genes. Few of the late genes (10.5%), 23% of the early genes, and none of the IE genes have +1 nucleosomes. However, some early genes, for example UL30, do seem to follow this rule (Fig. 5A). Nevertheless, these +1 nucleosomes do not seem to be involved in nucleosome phasing as they are often followed by a NFR. It is not clear why the E genes should have twice the level of +1 nucleosome containing genes as the L genes and it will be interesting to compare this number at other times of infection.

### There are no nucleosomes on the “Joint” region of the genome

The HSV-1 genome consists of 2 segments, which can isomerize relative to each other giving 4 different genome conformations. The sequence at which this occurs is called the “Joint”. Virion DNA is linear and consequently has one joint. After entry into the cell nucleus, at the beginning of the infection cycle, the ends of the linear genome join to form a second copy of the “joint”, which is identical to the first joint sequence [8,68]. Interestingly, these joint regions appears to be devoid of nucleosomes, and the gene closest to the “joint” ICP 34.5 (a late gene) has only one nucleosome—perhaps due to its proximity to the “joint”. Interestingly this nucleosome is not a phasing nucleosome but present on the middle of the gene.

### Chromatin on other herpesviruses genomes

Cytomegalovirus (CMV) is a lymphotropic herpesvirus that grows slowly in tissue culture cells. Studies of CMV chromatin assembly suggest that there are distinct early and late states of CMV chromatin that differ markedly in their level of chromatin (6 fold more at 48 h P.I., compared to 2 h P.I. [76]. These studies also note that not all CMV genomic regions are affected equally by the late increase. A temporal switch in chromatin organization has been revealed for CMV by Zalckvar et al., who proposed that the chromatin change was governed by a major IE protein [55]. It will be interesting to determine in future studies if there is any difference in HSV-1 chromatin between early and late stages of infection. Clearly there is a difference between lytic infection and latency [11,12,58]. Studies on EBV have revealed that there may be higher order chromatin structures present on viral DNA including DNA loops, chromatin domains, and insulators, which may play an important role in programming gene expression [77]. Although EBV is useful for studies on latent chromatin it is less useful than HSV-1 for studies on chromatin in lytic replication and the transition to latency.

### Conclusions

Our data show that the HSV-1 genome is partially covered by nucleosomes at 6h PI in SY5Y tissue culture cells thus explaining the lack of MNase partial digest band pattern. The distribution of nucleosomes in the population of viral genomes is not random because distinct regions of the genome exist where we detect nucleosomes and there are other regions that are nucleosome free (NFR). This is similar to cellular genes which are being expressed where NFR regions are also seen. Most viral gene promoters are nucleosome free, probably because they are highly active at 6h post infection. Cell genes usually have a +1 nucleosome at the start of transcription that acts to position other nucleosomes on the gene, however, this is not often seen in HSV-1 genes, and when it is, it does not seem to be related to phasing of nucleosomes. Although no statistical significance, there is a trend to less nucleosomes on immediate early genes compared to early and late genes. Furthermore, nucleosome density does not seem to correlate to GC levels as in cell genes. In fact, the “Joint”, a high GC region of the genome, is NFR. The Ori_S,_ in a high GC region, is nucleosome free and the Ori_L_ is in a relatively low GC region and is also essentially nucleosome free. Generally, our findings suggest there are different rules defining the positioning of nucleosomes on the HSV-1 genome compared to those for cell genomes. These findings do not support a statistical positioning model for HSV-1 nucleosomes from a barrier near the promoter involving some aspects of transcriptional initiation by RNA Polymerase II [68]

## Supporting Information

S1 Fig Genome Nucleosome Map. Nucleosome assignments covering whole genome.
(PDF)

S2 Fig Gene Nucleosome Table. Calculation of nucleosome saturation and GC.
(XLSX)
