# Supplementary figures and images for: Genome Wide Nucleosome Mapping for HSV-1 Shows Nucleosomes Are Deposited at Preferred Positions during Lytic Infection
Source: PLoS One. 2015 Feb 24;10(2):e0117471. doi: 10.1371/journal.pone.0117471 (PMC4339549; doi:10.1371/journal.pone.0117471)

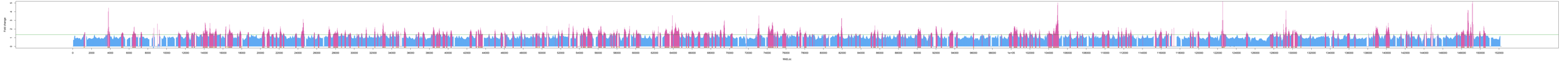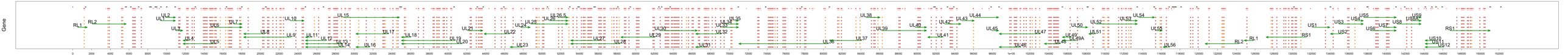

Supplement: S1 Fig — Nucleosome assignments covering whole genome. (PDF) [file pone.0117471.s001.pdf]
